# Effect of *Ducrosia flabellifolia* and *Savignya parviflora* Extracts on Inhibition of Human Colon and Prostate Cancer Cell Lines

**DOI:** 10.3390/cimb43030107

**Published:** 2021-10-10

**Authors:** Youssef Saeed Alghamdi, Osama Moseilhy Saleh, Nada Alqadri, Mutaib Mosaued Mashraqi, Omar Bahattab, Nabil Saied Awad

**Affiliations:** 1 Department of Biology, Turabah University College, Taif University, Taif 21995, Saudi Arabia; Ysghamdi@tu.edu.sa (Y.S.A.); naqadri@tu.edu.sa (N.A.); 2 Natural Products Research Department, National Centre for Radiation Research and Technology (NCRRT), Egyptian Atomic Energy Authority, Cairo 9621, Egypt; osama.meselhy@must.edu.eg; 3 College of Biotechnology, Misr University for Science and Technology, Giza 12563, Egypt; 4 Department of Clinical Laboratory Sciences, College of Applied Medical Sciences, Najran University, Najran 61441, Saudi Arabia; mmmashraqi@nu.edu.sa; 5 Department of Biology, Faculty of Science, University of Tabuk, Tabuk 71497, Saudi Arabia; Obahattab@ut.edu.sa; 6 Department of Genetics, Faculty of Agriculture and Natural Resources, Aswan University, Aswan 81528, Egypt

**Keywords:** *Ducrosia flabellifolia*, *Savignya parviflora*, anti-cancer, colon cancer, prostate cancer, gene expression

## Abstract

The goal of this study was to investigate whether *Ducrosia flabellifolia* and *Savignya parviflora* methanol extract the have effect on colon and prostate cancer cell lines. Analysis of total content of phenolics and flavonoids of each plant extract was carried out. Cytotoxic effect, cell cycle analysis, induction of apoptosis and gene expression of *Bcl-2* and *Bax* genes were studied. Obtained results indicated that, the plant extracts exhibit growth inhibition of used cancer cell lines and induced apoptosis as well as arresting of cell cycle. At the molecular level, changes in gene expression were detected via qPCR and confirmed by western blotting. The exhibited anticancer potentialities of plant extracts against utilized cancer cell lines are due to its containing bioactive compounds. Further detailed isolation, fractionation and characterization of bioactive compounds are needed.

## 1. Introduction

Worldwide, cancer is one of the most serious health problems [1]. Overcoming this threat requires many health challenges worldwide. Colorectal, lung and breast cancers are the most common types of cancer worldwide [2]. Young males with colorectal cancer have an increased risk of cancer prostate [3]. Colon cancer increasing within a group of less than 50 years prostatic cancer patients [4].

The ineffectiveness of clinical cancer rehabilitation approaches, such as chemotherapy, radiotherapy, surgery, and immunosuppression are manifested with high mortality and morbidity. This has created a necessary for promising, effective and safe compounds for cancer prevention and treatment [5]. Thus, herbal remedies have been reborn as nutritional and botanical preparations. Many medicinal plants are used directly as botanicals of medical importance. Medicinal plants are considered as one of a great resource of chemicals with potential therapeutic effects [6].

Effects of plants such as the active components of antitumor compounds have been shown to increase the effects of drugs, where drugs such as vinblastine, vincristine, toxol and camptothecin have improved the chemotherapy of some cancers [7]. Some species, such as *Convolvulus* sp. and *Astragalus* sp. have the potential to induce apoptotic cell death against lung and colon cancer cell lines [8].

The Arabian Gulf region have several species of *Ducrosia* [9]. Coumarin is one of the various phytochemicals found in it [10]. Active components of *Ducrosia flabellifolia* have been collected and used as an anticancer for various cell lines and have been demonstrated to reduce the likelihood of tumor burden [11]. *D. flabellifolia* contains many active ingredients such as Ducrosin B, which shows excellent cytotoxicity against human ovary; SKOV-3 and colon; HCT-116 cell lines [12]. Another active ingredient though; furocoumarin has activity against human hepatoma; HepG2 cell lines [13].

The ethanolic extract of *S. Parviflora* has abundant useful active ingredients, including anti-inflammatory, anti-ulcerogenic and antioxidant agents [14]. Ethanol extract of *S. Parviflora* at concentrations of 25 and 50 µg/mL inhibits RAW264.7 cell line growth and is might be promising for antitumor use as reported [15]. Inhibition of metalloproteinase 2 activity, cell growth inhibition, induction of apoptosis, cell migration inhibition, signal pathway of survival suppression and invasion of the HeLa cervical cancer cell lineage have been documented after treatment with *Kaempferia parviflora* extract [16]. They suggest that the herb contains active compounds that suppress EGF-based signal transduction pathways and then inhibit tumor progression and induce cancer cell death. *Coniferia parviflora* extract is a new candidate for an ovarian cancer chemotherapeutic agent in response to cell proliferation, cell migration and support apoptotic cell death induction in SKOV-3 cells [17].

This study aimed to in vitro assessment of the effectiveness of *D. flabellifolia* and *S. parviflora* methanolic extracts as natural herbs against prostate and colon cancer cell lines. Moreover, the molecular mechanism behind antitumor activity through these two extracts was investigated by cell cycle analysis, apoptosis analysis, gene expression analysis using qPCR and Western blotting assays.

## 2. Materials and Methods

### 2.1. Research Design and Statistical Analysis

To examine the antiprolifrative effect of each plant extract, the cancer cells treated with different concentrations of plant extract to identify the IC50 in comparison with un-treated cells.

IC50 had been used to explore the ability of plant extract on the induction of apoptosis, cell cycle arrest, gene expression among treated and untreated cells.

Students’ *t*-test was performed to assess the significant differences between treated and untreated cells. Statistical analysis was performed using SPSS version 22.0 software (IBM, Chicago, IL, USA). The *p*-value < 0.05 is statistically significant and the *p*-value < 0.01 is highly significant.

### 2.2. Plant Materials

The plant samples were collected and dried at Taif, West Region, Saudi Arabia. Samples of dried plants were ground into powder. Plant species have been identified and certified as *D. flabellifolia* and *S. parviflora*, which belong to the families *Apiaceae* and *Brassicaceae*, respectively.

### 2.3. Preparation of Methanolic Extract

Dry plant materials were used to prepare the methanolic plant extract via the Soxhlet extraction procedure. The methanolic extracts were evaporated to dryness and concentrated under pressure at temperatures; 40 to 50 °C in a rotary evaporator. The extracts have been collected and stored in airtight and dark bottles until use.

All the following experimental analyses were carried out in three replicates

### 2.4. Analysis of the Total Content of Phenolics, Flavonoids and Antioxidant Effect of Plant Extracts

Total content of phenolics (TPC) and flavonoids (TFC) were estimated in *D. flabellifolia* and *S. parviflora* plant extracts using a colorimetric assay according to [18] based on procedures described by [19] and [20], respectively. The total phenolic compounds were illustrated as mg/g gallic acid equivalent (GAE). Total flavonoid content (mg/g) was measured using a calibration curve of quercetin and illustrated as mg quercetin equivalents. The antioxidant activity of the extracts collected has been studied according to the methods described by [21] with minor modifications as applied by [22]. Briefly, methanolic extracts of *D. flabellifolia* and *S. parviflora* in various concentrations (100–500 μL) were mixed separately with 2.5 mL of 0.2 mM PBS (pH 7.4) and 2.5 mL of potassium ferricyanide (1% *w*/*v*). This mixture was incubated at 500 °C for 20 min. Then, 2.5 mL of trichloroacetic acid (10% *w*/*v*) was added and centrifuged at 3500 rpm for 8 min, followed by 2.5 mL of distilled water and later 0.5 mL of ferrous chloride (0.1% *w*/*v*). The absorbance at 700 nm was estimated. As a positive reference standard, ascorbic acid was utilized.

### 2.5. Cell Lines and Cell Cultures

The HCT 116 and PC3 cancer cell lines were used. The used cell lines were purchased from VACSERA, Egypt. The RPMI 1640 medium (Gibco-BRL, Carlsbad, CA, USA) was used to culture the cells. Medium was enhanced with FBS (Sijixin Inc., Dalian, China) and 1% penicillin–streptomycin mixture (Invitrogen, Grand Island, NY, USA) and incubated at 37 °C in CO_2_ incubator with 5% CO_2_.

### 2.6. In Vitro Cytotoxicity Assay

For the cytotoxicity assay, 1 × 10^5^ cells/mL (100 μg/well) were seeded in 96-well tissue culture plates and incubated at 37 °C for 24 h to produce a complete monolayer sheet. Using methanol extracts at different concentrations; 4–100 ug /mL, each plant was incubated at 37 °C with and without extract. After 72 h of incubation, cytotoxicity was assessed using MTT assay as reported by [23]. The cells were washed with 1 × phosphate buffer saline and incubated for 2–3 h with MTT (3-[4,5-dimethylthiazol-2-yl]-2,5-diphenyltetrazolium bromide) solution (50 μL of 0.5 mg/mL in RPMI 1640 without phenol red; Sigma). Then, MTT solution was removed and formazan crystals were dissolved in 75 μL of isopropanol: HCl (*v*/*v* 1:0.04) mixture. The viable cells were detected spectrophotometrically by the absorption at 570 nm for both treated and untreated cells in addition of wells without sample containing cells as blanks. Measurements were carried out and the IC50 was graphically determined. The percentages of viable cells were calculated as follows: % cell viability = A570 of treated cells/A570 of control cells × 100.

### 2.7. Apoptosis Detection

Annexin V-fluorescein isothiocyanate (FITC)/propidium iodide (PI) staining was used to estimate apoptotic cells after treatment of HCT and PC3 cells with IC50 concentration of each plant extract in comparison with untreated cells.

### 2.8. Cell Cycle Analysis

Cell cycle analysis of plant extracts treated, and untreated cells was carried out using flow cytometry (BD FACS Array Bioanalyzer) to detect the cellular phase in which the cell cycle has been arrested.

### 2.9. Detection of Expression Levels of Bax and Bcl-2 Genes

Among treated with IC50 and untreated HCT and PC3 cells, changes in mRNA levels of apoptotic-related genes *Bax* and *Bcl-2* were analyzed using qPCR as previously described [24]. HCT-116 and PC3 cells were treated with IC50 concentrations for 48 h. Total RNA was carried out according to triazole reagent manufacturer’s instructions. 

### 2.10. Real-Time Quantitative PCR (qPCR)

One μg of RNA was reverse transcribed to first-strand cDNA. The obtained cDNA was amplified to examine the expressions of *Bcl-2* and *Bax* genes. An internal control *β-actin* was utilized as a standard for the real-time PCR reaction. Sequences of forward and reverse primers for genes used in this study are shown in Table 1.

The thermocycle program for cDNA amplification was carried out as follow: 95 °C for 35 s followed by 40 cycles of denaturation (95 °C for 5 s), annealing at 58 °C for 10 s (*β-actin*) and 55 °C for 10 s (*Bax* and *Bcl-2*), and extension at 72 °C for 30 s. A non-template control had been done using water instead of the cDNA template. All PCR amplified products were electrophoresized with DNA ladder (100 bp) (Fermen-tas, USA) in 2.5% agarose gel. Ethidium bromide was used to visualize the gels using a UV trans-illuminator. Quantitative real-time (qRT-PCR) was used to quantify *Bcl-2*, *Bax* and *β-actin* mRNA levels. A real-time PCR kit (BIORAD iScript^TM^ One-Step RT-PCR Kit) using SYBR Green used to perform the quantitative RT-PCR. Diethyl pyro carbonate (DEPC) water was used as a negative control instead of the cDNA template. The results for *Bcl-2* and *Bax* mRNA expression exhibited in relation to the expression of *β-actin*. The PCR products were determined by melting curve analysis for each primer pairs to specify the amplification. The 2^ΔΔCT^ method was used to analyze the obtained data.

Target mRNA level results were generalized against *β-actin* mRNA. Results were presented as fold change (RFC) relative to negative control.

### 2.11. Western Blotting Analysis

Western blotting method has been used to assess the effect of tested plant extracts on the translation level of the *Bax* and *Bcl-2* genes as described previously by [25,26]. 

## 3. Results

### 3.1. Analysis of the Total Content of Phenolics, Flavonoids and Antioxidant Effect of Plant Extracts 

**Figure 1:**
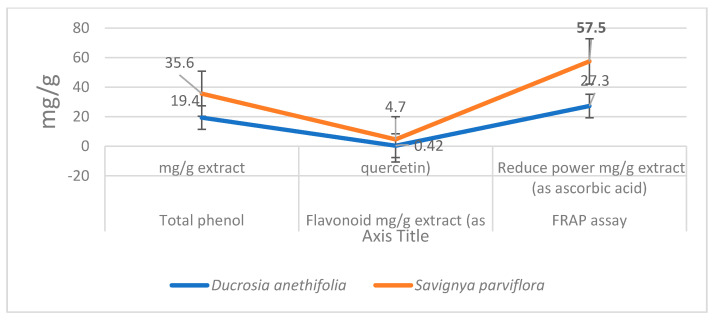
Analysis of total content of phenolics and flavonoids and their antioxidant power.

Among studied plant extracts, the total phenolic content (TPC) and total flavonoid content (TFC) were measured. Obtained results showed that, *S. parviflora* extract had higher amounts of TPC and TFC than *D. flabellifolia* as in the case of antioxidant power (Figure 1). 

### 3.2. Cytotoxic Effects

The cytotoxic effect of methanol extract of *S. parviflora* and *D. flabellifolia* on the growth of HCT-116 and PC3 cell lines were separately examined by MTT assay. Dose dependent response was obtained between the range 0.4 and 100 µg/mL for plant extracts and Staurosporine (standard control) decreasing number of viable cells with an increasing concentration of plant extracts as well as Staurosporine were noted. Calculation of IC50 value was carried out (Table 2).

**Figure 2:**
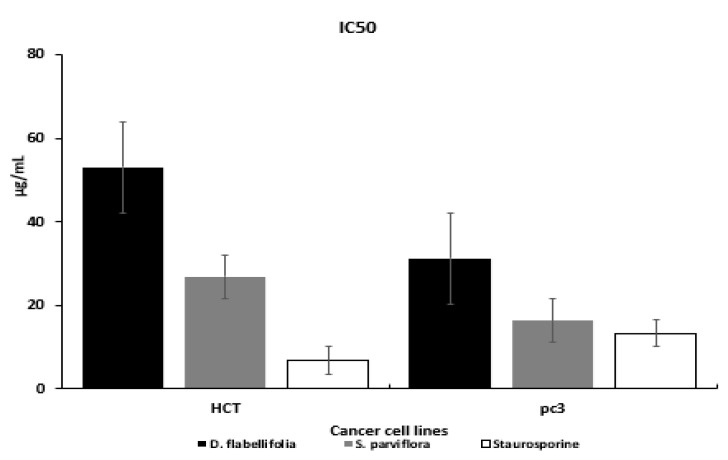
Inhibition concentration of *D. flabellifolia* and *S. parviflora* against HCT-116 and PC3 cell lines.

The susceptibility of cells to the examined plant extracts was characterized by IC50 (Table 3) and (Figure 2). The IC50 values of *S. parviflora* with HCT-116 and PC3 cell lines were (26.9 and 16.4 ug/mL respectively), which lowers than the IC50 values of *D. flabelli*-*folia* against the two cell lines. 

### 3.3. Apoptosis Detection

**Figure 3:**
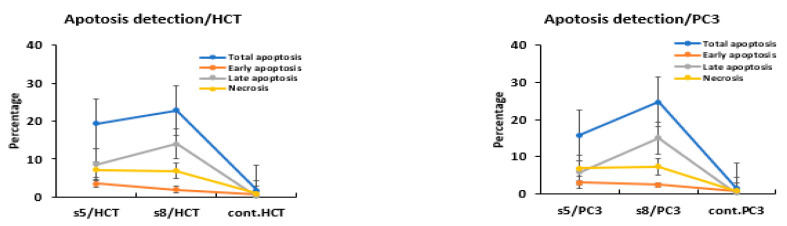
Effects of IC50 concentrations of *D. flabellifolia* (S5) and *S. parviflora* (S8) on the induction of apoptosis among HCT-116 and PC3 cell lines.

**Figure 4:**
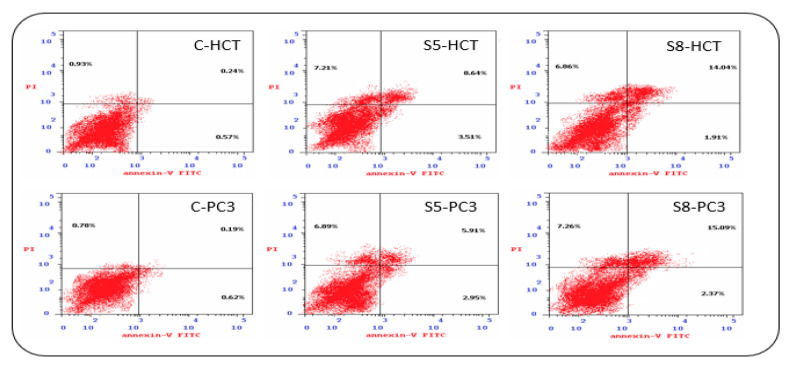
Induction of apoptosis among HCT-116 and PC3 cell lines in response to treatment with IC50 of *D. flabellifolia* (S5) and *S. parviflora* (S8) extracts in comparison with controls (C).

The extracts’ potentially to induce apoptosis was examined in HCT-116 and PC3 cancer cells using annexin-FITC/propidium iodide double staining cytometric assay. The cells were treated with IC50 of each extract for 48 h (Figure 3 and Figure 4). At IC50 concentrations, HCT-116 and PC3 cells underwent late apoptosis and induced mild necrosis.

### 3.4. Cell Cycle Analysis

**Figure 5:**
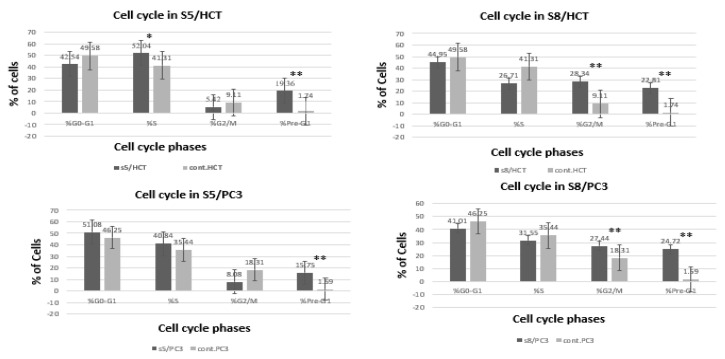
Cell cycle analysis of HCT-116 and PC3 cell lines in response to treatment with IC50 of *D. flabellifolia* (S5) and *S. parviflora* (S8) extracts in comparison with controls (C). Statistically significant indicated by * *p*-value < 0.05 and highly significant indicated by ** *p*-value < 0.01.

Cell cycle analysis was carried out in treated and untreated cancer cell lines. In response to treatment of the HCT-116 cell line with IC50 of *D. flabellifolia* extract, cells arrested significantly in the S and pre-G1 phases. In addition, PC3 cells were significantly arrested in the pre-G1 phase. On the other hand, treatment of HCT-116 and PC3 with IC50 of *S. parviflora* extract induced significantly arresting of the cell cycle at G2/M and pre-G1 phases (Figure 5).

### 3.5. Gene Expression Analysis

The gene expression level changes of *Bax* and *Bcl-2* genes in response to treatment with IC50 of *D*. *flabellifolia* and *S. parviflora* extracts were assessed by two different methodologies (qPCR and Western blotting).

**Figure 6:**
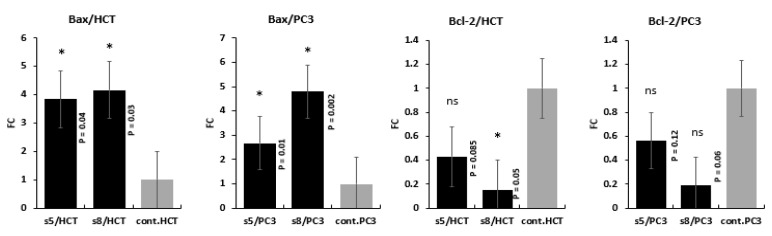
Changes in mRNA levels of *Bax* and *Bcl-2* genes among HCT-116 and PC3 cell lines in response to treatment with IC50 of *D. flabellifolia* (S5) and *S. parviflora* (S8) extracts in comparison with controls (C). * *p*-value < 0.05; ns = non significant.

**Figure 7:**
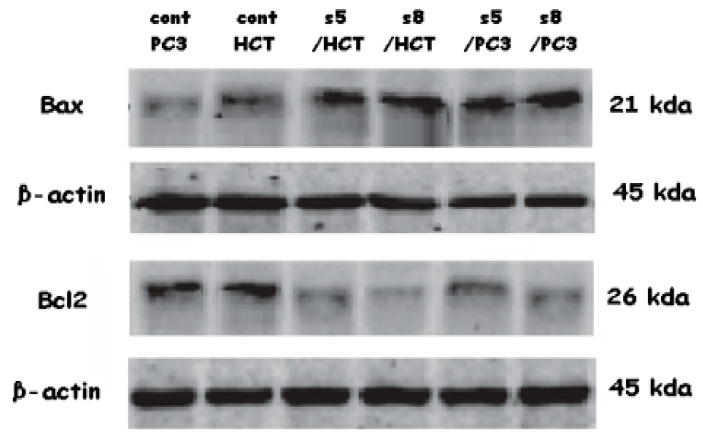
Changes in protein levels of *Bax* and *Bcl-2* genes among HCT-116 and PC3 cell lines in response to treatment with IC50 of *D. flabellifolia* (S5) and *S. parviflora* (S8) extracts in comparison with controls (C).

**Figure 8:**
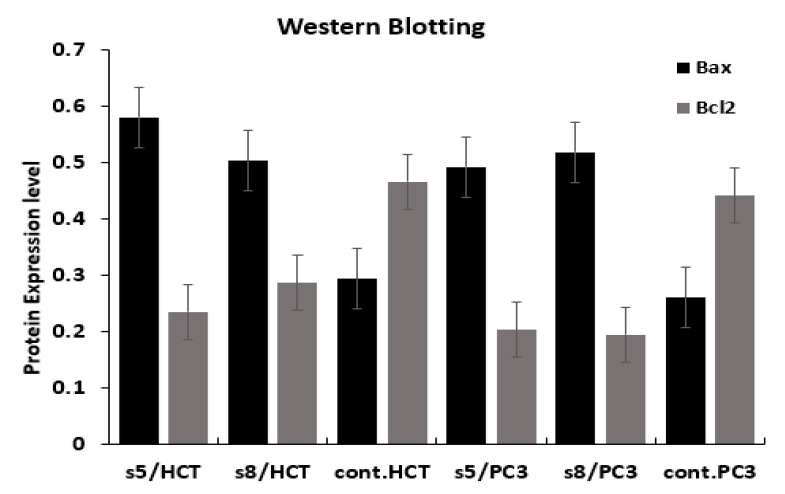
Western blotting analysis showed changes in protein levels of *Bax* and *Bcl-2* genes among HCT-116 and PC3 cell lines in response to treatment with IC50 of *D. flabellifolia* (S5) and *S. parviflora* (S8) extracts in comparison with controls (C).

Results of qPCR indicated that, treatment of HCT-116 and PC3 with IC50 of *D*. *flabellifolia* and *S. parviflora* extracts leads to upregulation of *Bax* gene and downregulation of *Bcl-2* gene (Figure 6). Expression level of *Bax* gene in both plant extract with the two utilized cell lines was upregulated significantly. *Bcl-2* gene expression level was downregulated significantly due to treatment of HCT with *S. parviflora* and not significant in treatment of HCT with *D. flabellifolia*. In the PC3 cell line, no significant effects were detected of both *S. parviflora* and *D. flabellifolia* extracts on *Bcl-2* gene expression. Regards to confirmation of the changes occurred in gene expression levels in response to treatments, the protein level of *Bax* and *Bcl-2* genes were measured via western blotting technique. The obtained results were consistent with Real time PCR data (Figure 7 and Figure 8).

## 4. Discussion

Cancer is considered as one of the first deadly diseases. There are many cancer treatments that depend on different methods of treatment. But it is established that all these treatments face many challenges such as adverse effects and side effects [27]. Hence, there has been a wide-ranging interest in discovering effective anticancer substances that avoid many of the challenges. Wild plants are considered an influential source in the discovery of many natural substances with anticancer abilities. Therefore, there is an interest in researching and discovering more wild plants that may have natural substances with which it is possible to develop new safe and high quality treatments against cancer [28].

The obtained results of our study showed that the methanolic extracts of both plants have anticancer effect against both colon and prostate cancer cell lines. This is what was shown by the results for cytotoxic effects and IC50 values (Table 2 and Table 3, Figure 2). From these values, the *S. parviflora* has a higher anticancer capacity than the *D. flabellifolia*. When the IC50 value is less 20 μg/mL, 20 to 100 μg/mL or higher 100 μg/mL, the US National Cancer Institute considers the cytotoxicity standard for raw extract to be active, moderately active or inactive respectively [29]. These results can be interpreted based on the analysis of the total content of phenolics, flavonoids and antioxidant activity (Figure 1) which showed that the total content of phenolics and flavonoids of *S. parviflora,* as well as antioxidative power, was higher than that of the *D. flabellifolia* [30,31,32,33]. It showed that the cytotoxic effect of the extracts of these plants was higher on prostate cancer cell (PC3) than on colon cancer cell (HCT-116). The antiproliferative effects observed for both plant extracts against the two cancer cells used may be due to the oxidant effect of their phytochemical components. Plant substances containing phenolic compounds exert their antiproliferative effects through increase oxidant stress in cancer cells by inhibiting ROS-scavenging systems, inactivating pro-survival signals, activating apoptosis related signals, inducing DNA damage and inhibiting signaling favorable pathways to cancer cell growth [34]. To investigate the mechanism behind the cytotoxic effects of these plant extracts against colon and prostate cancer cell lines, flow cytometry was used to test the apoptosis events and cell cycle analysis. Our results indicated both plant extracts were able to arrest the cell cycle at different phases (Figure 5). It is reported that flavonoids and phenols can arrest the cell cycle in different cancer cells [35,36,37]. The results indicated that both plant extracts under study can induce apoptosis as late apoptosis and arresting the cells in different cell cycle phases (Figure 3 and Figure 4). Similarly, many natural agents exhibit anticancer or cancer protection properties by inducing apoptotic pathways in transformed cells in the process of carcinogenesis. [38,39]. In the present study, the expression of two mitochondrial apoptosis related genes (*Bax* and *Bcl-2*) was measured at transcription and translation levels. Gene expression study indicates upregulation of *Bax* and downregulation of *Bcl-2* (Figure 6, Figure 7 and Figure 8). These results suggest that *S. parviflora* and *D. flabellifolia* bioactive phytoconstituents induce apoptosis in HCT-116 and PC3 cells by mechanisms related to the mitochondria-based pathway. This is consistent with the reported results [40].

Results showed that *S. parviflora* and *D. flabellifolia* extracts have a significant anti-proliferative effect on HCT-116 and PC3 cells by inducing apoptosis in a significant way. The obtained results showed that the *S. parviflora* and *D. flabellifolia* extracts exerted a significant anti proliferative effect on the HCT-116 and PC3 cells by inducing apoptosis. The obtained results showed that the *S. parviflora* and *D. flabellifolia* promotes apoptosis which was achieved by the pro-apoptotic and anti-apoptotic members *Bax* and *Bcl-2* genes, respectively [41]. Although the antioxidant activity of phytochemicals is well known, they exhibit pro-oxidant activities under certain conditions at high doses, they produce ROS, with the oxidation of all hydroxyl-forming groups increasing radical production occurring the presence of metal ions [42]. cl-2 protein, as the main regulator of apoptosis, promotes cell survival by inhibiting factors that activate caspases [43], or by controling apoptosis, despite its active functional antagonism through the formation of heterodimers with other Bcl-2 family members. Bax, a pro-apoptotic member, also binds to the anti-apoptotic protein Bcl-2 and thus acts by inhibiting the action of Bcl-2 to terminate apoptosis. Moreover, induction of Bax was also observed to enhance the release of cytochrome c from mitochondria, which ultimately leads to apoptosis [44]. It was suggested that both Bcl-2 family of proteins (Bax and Bcl-2) play pivotal roles in *S. parviflora* and *D. flabellifolia* induced apoptosis. Thus, the results indicated that the observed upregulation of *Bax* and corresponding downregulation of *Bcl-2* genes might be one of the crucial mechanisms that induce which *S. parviflora* and *D. flabellifolia* apoptosis in HCT-116 and PC3 cells. *S. parviflora* and *D. flabellifolia* extracts permanently interfered with the cell cycle in vitro (Figure 5), significantly arresting cells in different phases indicating the pro-apoptotic activity of the extracts. The results of this research and others, which show that Bcl-2 family members have cell cycle inhibitory functions, now provide an explanation for how *Bcl-2* downregulation and of *Bax* upregulation may play a role in tumor progression [45,46].

## 5. Conclusions

Antiproliferative potentiality of *S. parviflora* and *D. flabellifolia* methanolic extract against HCT-116 and PC3 cancer cells was detected. Both plant extracts exhibited proliferation inhibition of HCT-116 and PC3 cancer cells. Regards to the IC50, the *S. parviflora* was lower than the IC50 of *D. flabellifolia*. Results of cell cycle analysis and induction of apoptosis pointed that the antiproliferative potentiality of studied extracts was due to the induction of late apoptosis and arresting cells at different cell cycle phases. The level of gene expression was changed due to treatments with each plant extract IC50. The *Bax* gene expression was upregulated while *Bcl-2* gene expression was downregulated. Results showed that the extracts contained antiproliferative bioactive compounds that require further larger and detailed studies focused on fractionation and characterization of these bioactive compounds, as well as study the mode of action of such compounds at different levels.

## Figures and Tables

**Table 1:** The sequences of the primers used were as follows.

	Gene	Primer Sequence		Annealing Temperature
		Forward	Reverse	
1	Bcl-2	5′-CCTGTGGATGACTGAGTACC-3′	5′-GAGACAGCCAGGAGAAATCA-3′	55 °C
2	Bax	5′-TTCCGAGTGGCAGCTGAGATGTTT-3′	5′-TGCTGGCAAAGTAGAAGAGGGCAA-3′	55 °C
3	β-actin	5′-GTGACATCCACACCCAGAGG-3′	5′-ACAGGATGTCAAAACTGCCC-3′	58 °C

**Table 2:** In vitro cytotoxic activity of *D. flabellifolia* and *S. parviflora* against HCT-116 and PC3 cell lines.

Concentration (µg/mL)	% of Viability			
	*D. flabellifolia*		*S. parviflora*	
	HCT-116	PC3	HCT-116	PC3
100	45	43	40	40
25	57	52	52	45
6.3	64	60	59	56
1.6	73	69	71	68
0.4	84	78	77	71

**Table 3:** Inhibition concentration IC50 of *D. flabellifolia* and *S. parviflora* against HCT-116 and PC3 cell lines.

No.	Sample	Cytotoxicity	
	Code	IC50 µg/mL	
	Code	HCT	PC3
1	*D. flabellifolia*	53.1 ± 2.98	31.3 ± 1.7
2	*S. parviflora*	26.9 ± 1.51	16.4 ± 0.89
3	Staurosporine	6.9 ± 0.39	13.4 ± 0.73

## Data Availability

Not applicable.
